# Quantitative Release Assessment of *mcr*-mediated Colistin-resistant *Escherichia Coli* from Japanese Pigs

**DOI:** 10.14252/foodsafetyfscj.D-20-00004

**Published:** 2020-06-26

**Authors:** Kohei Makita, Yuri Fujimoto, Nami Sugahara, Takeshi Miyama, Masaru Usui, Tetsuo Asai, Michiko Kawanishi, Manao Ozawa, Yutaka Tamura

**Affiliations:** 1 Veterinary Epidemiology Unit, Division of Health and Environmental Sciences, Department of Veterinary Medicine, School of Veterinary Medicine, Rakuno Gakuen University, 582 Bunkyodai Midorimachi, Ebetsu069-8501, Japan; 2 Food Hygiene Unit, Division of Health and Environmental Sciences, Department of Veterinary Medicine, School of Veterinary Medicine, Rakuno Gakuen University, 582 Bunkyodai Midorimachi, Ebetsu 069-8501, Japan; 3 Department of Applied Veterinary Sciences, United Graduate School of Veterinary Sciences, Gifu University, 1-1 Yanagido, Gifu 501-1193, Japan; 4 National Veterinary Assay Laboratory, 1-15-1 Tokura, Kokubunji 185-0003, Japan

**Keywords:** colistin, *mcr*, quantitative risk assessment

## Abstract

Colistin is a critically important antibiotic for humans. The Japanese government withdrew colistin growth promoter and shifted therapeutic colistin to a second-choice drug for pigs in 2017. A quantitative release assessment of *mcr*-mediated colistin-resistant *Escherichia coli* (*E. coli*) in Japanese finisher pigs was conducted under the World Organisation for Animal Health (OIE) risk assessment framework. Input data included colistin resistance and *mcr-1-5* test results for *E. coli* isolates in the Japan Veterinary Resistance Monitoring System (JVARM), postal survey results regarding indication disease occurrence and colistin use by swine veterinarians in 2017 and 2018, and colistin resistance and *mcr* monitoring experiments at four pig farms in 2017-2018. An individual-based model was developed to assess the risk: the proportion of Japanese finisher pigs with *mcr-1-5*-mediated colistin-resistant *E. coli* dominant in the gut on an arbitrary day. Before implementing risk management measures, the risk was estimated to be 5.5% (95% CI: 4.2%-10.1%). At 12 months after stopping colistin growth promoter, the proportion of pigs with plasmid-mediated colistin-resistant *E. coli* declined by 52.5% on the experiment farms (95% CI: 8.7%-80.8%). The probability of therapeutic colistin use at the occurrence of bacterial diarrhea declined from 37.3% (95% CI: 30.3%-42.5%) in 2017 to 31.4% (95% CI: 26.1%-36.9%), and that of edema disease declined from 55.0% (95% CI: 46.0%-63.7%) to 44.4% (95% CI: 36.9%-52.0%). After risk management implementation, the risk was estimated to have declined to 2.3% (95% CI: 1.8%-4.3%; 58.2% reduction). Scenario analyses showed that pen-level colistin treatment effectively reduces the risk from 5.5% to 4.7% (14.5% reduction), an effect similar to stoppage of therapeutic colistin (16.4% reduction to 4.6%).

## 1. Introduction

Colistin is a critically important antibiotic called an antibiotic of last resort^1^^)^ in light of the rapid global rise of multi-drug-resistant Enterobacteriaceae. Colistin sulfate, a polypeptide antibiotic, has been used in Japan since the 1950s for the treatment of gram-negative gastrointestinal infections and as a feed additive to promote healthy development in food-producing animals (cattle, swine, and chickens)^2^^,^^3^^)^. In human medicine, the use of injection formulas, which had been suspended due to the frequent adverse effects such as renal dysfunction, was re-approved in 2015 in response to the global rise of multi-drug-resistant gram negative bacterial infections^3^^)^.

Polymyxins (polymixin B and colistin) modify the lipopolysaccharide (LPS) of gram-negative bacteria by decreasing the negative charge of the lipid A moiety of LPS. Chromosomal colistin resistance is caused by the activation of two-component systems involving PhoP/PhoQ and PmrA/PmrB via mutation, which results in the overexpression of LPS-modifying genes^4^^)^. Prior to 2015, when a mobile colistin-resistance gene, *mcr-1*, was reported in China^5^^)^, this was the only known mechanism of colistin resistance. The *mcr* gene, which is harbored on a plasmid, can be transmitted between bacteria, which poses a significant threat to humans, as important Enterobacteriaceae pathogens such as multi-drug-resistant *Pseudomonas aeruginosa* (MDRP), multi-drug-resistant *Acinetobacter* (MDRA), and carbapenem-resistant Enterobacteriaceae (CRE) can acquire colistin resistance as well. Since the first discovery of *mcr-1* in China, identification of different *mcr* genes has continued globally, and as of January 2020, *mcr-1* to *-10* have been reported^6^^,^^7^^,^^8^^,^^9^^,^^10^^)^. In Japan, a high prevalence of *mcr-1* (30.0%), *-3* (8.3%), and *-5* (28.3%) was reported among 120 isolates from diseased pigs^11^^)^, and a low proportion (1.9%, 39/2052 isolates) of *mcr-1* and the absence of *mcr-2* was reported among healthy pigs^2^^)^.

The Food Safety Commission of Japan (FSCJ) immediately conducted a qualitative risk assessment for colistin resistance after the discovery of *mcr-1*^5^^)^, which determined the risks of release, exposure, and consequence to be medium, low, and high, respectively^3^^)^. Based on these risk assessment results, reported in January 2017, the Ministry of Agriculture, Forestry, and Fisheries (MAFF) of Japan announced a stoppage of market sales of colistin growth promoter and shifted therapeutic colistin from a first-choice to second-choice drug in December 2017. The actual withdrawal of colistin growth promoter from the market and the shift to second-choice drug took effect on July 1, 2018, and April 1, 2018, respectively.

The objectives of this study were to quantitatively assess the current risk of producing finisher pigs harboring *mcr-*mediated colistin-resistant *Escherichia coli *(*E. coli*) at farms just before sending the animals to the slaughterhouse and estimate the effects of potential control measures, including those already implemented via the risk management measures instituted by the MAFF.

## 2. Materials and Methods

### 2.1. Framework of the Risk Assessment

This study employed an World Organisation for Animal Health (OIE) risk assessment framework^12^^)^, which comprised a release (entry) assessment, exposure assessment, and consequence assessment. Release, in this case, is the use of colistin in pigs and selection of *mcr*-mediated colistin-resistant *E. coli*; exposure refers to a consumer ingesting *mcr*-mediated colistin-resistant *E. coli* due to consumption of pork derived from pigs administered colistin; and consequence refers to the effect of treatment failure when using colistin to treat an illness caused by *mcr*-mediated colistin-resistant bacteria, including those that obtained *mcr *genes via plasmids from *mcr*-mediated colistin-resistant *E. coli*. Among these steps, this study focused on the release assessment.

The risk was defined as the proportion on a given day of Japanese finisher pigs with *mcr*-mediated colistin-resistant *E. coli* dominating the gut, among all Japanese finisher pigs just before sending the animals to the slaughterhouse. Dominance in the gut by *mcr*-mediated colistin-resistant *E. coli* was defined as a concentration of *mcr*-mediated colistin-resistant *E. coli* in the gut higher than 10^5.08^ CFU/g, following setting of the cut-off point as described in the Results section. Release was defined as both the use of colistin as a feed-additive growth promoter and therapeutic use of colistin, including metaphylaxis, mass medication of healthy animals when the disease of interest is present within the group/flock/herd^13^^)^, at an occurrence of either edema disease or bacterial diarrhea during the weaning period.

Colistin resistance in *E. coli* was defined as a minimal inhibitory concentration (MIC) of ≥ 4 μg/mL, according to the European Committee on Antimicrobial Susceptibility Testing breakpoints for Enterobacteriaceae, MIC > 2 μg/mL (http://www.eucast.org/clinical_breakpoints/). In Japan, the presence of *mcr*-*1*-harboring *E. coli* with an MIC of 2 μg/mL has been reported^2^^)^, and these bacteria were considered susceptible to colistin in our study.

As of January 2019, when a risk assessment was conducted for 1,315 *E. coli* isolates collected between 2006 and 2015, the Japan Veterinary Antimicrobial Resistance Monitoring System (JVARM) of the National Veterinary Assay Laboratory (NVAL), MAFF of Japan, had tested for *mcr-1* through *mcr-5* among *mcr-1* to *-10*. Of these, 59 isolates had an MIC ≥ 4 μg/mL, and 41 isolates (41/59, 69.5%) had either *mcr-1*, *-3*, or *-5*, suggesting the remaining 30.5% involved either chromosomal or other *mcr*-mediated resistance (no isolate had *mcr-4*). As our study defined plasmid-mediated colistin-resistant *E. coli* as those harboring *mcr-1* to *-5*, the results may underestimate the actual risk for *mcr*-mediated colistin-resistant *E. coli* dominating the gut of pigs in Japan.

### 2.2. Data Collection

The colistin resistance test results and detection of *mcr-1* through *-5* in *E. coli* isolates collected between 2006 and 2015, in which *mcr* genes were detected throughout the period, were provided by the JVARM. In-depth discussions regarding the mechanism of selection of plasmid-mediated colistin-resistant *E. coli* were conducted with the NVAL, university researchers examining antimicrobial resistance, and field swine veterinarians to ensure the quality of the risk assessment model in terms of both scientific and field aspects.

Two postal surveys were conducted providing the structured questionnaires (Table 1) to veterinarians belonging to the Japan Pig Veterinary Society in 2017 and 2018 in the same calendar period (November to December). The reason two surveys were conducted was to compare differences in the frequencies of edema disease and diarrhea in the weaning period and the probability of therapeutic use of colistin upon the occurrence of these diseases, between before and after the stoppage of feed-additive use of colistin as a growth promoter and the change in categorization of therapeutic colistin use from first to second choice in 2018. The representativeness of the responses was measured using Spearman’s correlation test for the numbers of farrow-to-finisher and reproduction farms for which information was collected in the postal surveys and the numbers registered in the Statistical Survey on Livestock of Japan by prefectures as of February 2017^14^^)^. The ethics of the questionnaire studies were assessed and approved for exemption from ethical examination on October 30, 2018, by Research Ethics Committee of the Rakuno Gakuen University.

**Table 1.** Contents of the 2017 and 2018 questionnaires for the pig veterinarians

Category	Content
***Attribute questions***	
Attributes of veterinarian	Affiliation; association/academic society
Supervising farms	The number of farrow-to-finisher and reproduction farms supervising, by prefecture
***Disease occurrence***	
Frequency of bacterial diarrhea during weaning period	The number of farms falling into size categories based on the number of sows (≤50, 51-100, 101-200, 201-500, and ≥501) and frequency categories (almost no occurrence, once in 2-3 years, once in 7-12 months, once in 4-6 months, once in 2-3 months, and more than once a month)
Proportion of weaning-period pigs having diarrhea at an occurrence	The allocation of percentages (summing to 100%) in terms of the proportion of weaning pigs on a farm affected (≤10%, 10.1-30%, 30.1-50%, 50.1-70%, 70.1-90%, and 90.1-100%) based on the current clinical situation. The allocation of 100% in total was requested for each farm size category based on the number of sows (≤50, 51-100, 101-200, 201-500, and ≥501)
Frequency of edema disease during weaning period	The number of farms falling into size categories based on the number of sows (≤50, 51-100, 101-200, 201-500, and ≥501) and frequency categories (almost no occurrence, once in 2-3 years, once in 7-12 months, once in 4-6 months, once in 2-3 months, and more than once a month)
Proportion of weaning-period pigs having edema disease at an occurrence	The allocation of percentages (summing to 100%) in terms of the proportion of weaning pigs on a farm affected (≤10%, 10.1-30%, 30.1-50%, 50.1-70%, 70.1-90%, and 90.1-100%) based on the current clinical situation. The allocation of 100% in total was requested for each farm size category based on the number of sows (≤50, 51-100, 101-200, 201-500, and ≥501)
Change in 2018 (only in the second questionnaire)	Changes in the frequencies of weaning period diarrhea and edema disease (increased, no change, decreased, don’t know)
***Colistin use***	
Feed additive use of colistin as a growth promoter (only in the first questionnaire)	Proportion of farms administering colistin-free feeds to weaning-period pigs in 2017 before stoppage
Probability of using therapeutic colistin	The probability of using therapeutic colistin at the occurrence of weaning-period diarrhea or edema disease
Change in the probability of therapeutic colistin use (only in the second questionnaire)	Change in the probability of therapeutic colistin use at the occurrence of weaning-period diarrhea or edema disease (increased, no change, decreased, don’t know)
***Colistin resistance cases***	
Probability of encountering an event of colistin resistance	The probability of encountering an event in which colistin is not effective when used
Change in the frequency of encountering an event of colistin resistance (only in the second questionnaire)	Change in the frequency of encountering an event in which colistin is not effective when used (increased, no change, decreased, don’t know)

### 2.3. Risk Assessment Model

An individual-based simulation model was developed using RStudio, version 1.1.456 (RStudio, Inc., Boston, MA, USA), to run in the statistics software R, version 3.5.1^15^^)^. The default setting models the feeding situation as of 2017, before stoppage of feed-additive use of colistin as a growth promoter. In total, 1,000 pig farms were generated in the model, representing Japanese farrow-to-finisher and reproduction farms in terms of the number of sows (212 small scale with 11-50 sows; 474 medium scale with 51-200 sows; and 314 large scale with 201-600 sows)^14^^)^. The numbers of reproduction and farrow-to-finisher farms in Japan as of 2017 February were 379, and 3,260, respectively; however, the output of the risk assessment is the proportion of finisher pigs with *mcr*-mediated colistin-resistant *E. coli* dominating the gut, and the risk can be correctly estimated. The number of sows in each of these 1,000 farms was randomly assigned by drawing from uniform distributions. In the model, all of the sows would give birth to 12 piglets, according to the expert opinions from swine medicine practitioners. All these piglets were monitored until finisher pigs. The model used probability distributions where necessary, and the types of distributions, parameters, and their sources are shown in Table 2 and Supplemental Table 9.

**Table 2.** Estimates of variables associated with the within- and between-farm prevalence of *mcr-1-5*-mediated colistin-resistant *E. coli* as used in the risk assessment

Variables	Distribution	Mean (median)	95% CI	Source
Proportion of *mcr-*mediated colistin-resistant *E. coli* dominant pigs in *mcr*-entered growth promoter feeding farms when therapeutic colistin is not used (*P_dom_gp_*)	Beta(12.851,28.739)	31.0% (30.6%)	18.0-45.6%	Farm experiment in 2017
Proportion of *mcr-*mediated colistin-resistant *E. coli* dominant pigs in *mcr*-entered growth promoter feeding farms when therapeutic colistin is used (*P_selected_gp_*)	Beta(22+1, 22-22+1)	95.9% (97.0%)	85.2-99.9%	Farm experiment in 2017
Proportion of *E. coli* isolates with any of *mcr-1* to *-8* in 2017 experiment (*P_mcr2017_*)	Point estimate, 16/90 isolates	17.8%*	-	Farm experiment in 2017
Proportion of *E. coli* isolates with any of *mcr-1* to *-8* in 2018 experiment (*P_mcr2018_*)	Point estimate, 6/90 isolates	6.7%*	-	Farm experiment in 2018
Reduction rate in the prevalence of pigs with *mcr*-mediated colistin-resistant *E. coli* (*Red_mcr_*)	Point estimate: 1-(1-(1-0.067)^3^^)^)/ (1-(1-0.178)^3^^)^) Stochastic: 1-(1-(1-Beta(6+1, 90-6+1))^3^^)^)/ (1-(1-Beta(16+1, 90-16+1))^3^^)^)	57.8%* 52.5%* (54.8%*)	- 8.7-80.8%	Farm experiment in 2017 and 2018
Proportion of *mcr-*mediated colistin-resistant *E. coli* dominant pigs in *mcr*-entered growth promoter non-feeding farms when therapeutic colistin is not used (*P_dom_nogp_*)	P_dom_gp_ * (1-*Red_mcr_*)	13.1% (12.9)	7.6-19.2%	Farm experiment in 2017 and 2018
Proportion of *mcr-*mediated colistin-resistant *E. coli* dominant pigs in *mcr*-entered growth promoter non-feeding farms when therapeutic colistin is used (*P_selected_nogp_*)	P_selected_gp_ * (1-*Red_mcr_*)	40.5% (40.9%)	36.0-42.2%	Farm experiment in 2017 and 2018
Proportion of *mcr-1-5*-mediated colistin-resistant *E. coli* positive samples in JVARM (*P_JVARM_*)	Beta(31+1,706-31+1)	4.5% (4.5%)	3.1-6.2%	JVARM
Proportion of *mcr-1-5-*harboring *E. coli* positive samples in JVARM including susceptible isolates (*P_JVARM2_*)	Beta(48+1,706-48+1)	6.8% (6.8%)	5.2-8.9%	JVARM
True farm level prevalence of *mcr-1-5*-mediated colistin-resistant *E. coli* (*P_TPF_*)	$P_{TPF}=\frac{P_{JVARM}}{P_{dom\_gp}}$	15.5% (14.8%)	8.6-26.5%	Logical
True farm level prevalence of *mcr-1-5-*harboring *E. coli* including susceptible isolates (*P_TPF2_*)	$P_{TPF2}=\frac{P_{JVARM2}}{P_{dom\_gp}}$	23.7% (22.6%)	13.9-40.1%	Logical

*Note: the point estimates are equivalent to the modes of beta distributions.

Out of 1,000 farms, pigs with *mcr*-harboring *E. coli* in the gut would be present in a proportion of farms, and the proportion of pigs with *mcr*-harboring *E. coli* dominating the gut in these farms was determined stochastically. In addition, 93% of pig farms administer feed-additive colistin growth promoter to weaning-period pigs, according to the above-mentioned questionnaire results. With or without the selection pressure of the growth promoter, bacterial diarrhea and/or edema disease would typically occur during 1 month of the weaning period with different probabilities between growth promoter–using and non-using farms, and veterinarians would use therapeutic colistin by adding it to the feed tank of the pigsty at a certain probability.

**Fig. 1.**
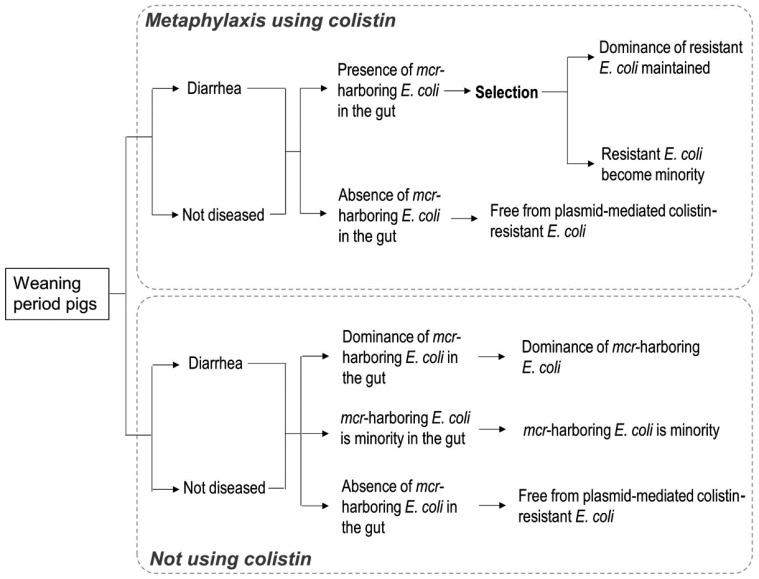
Flowchart for plasmid-mediated colistin-resistant *E. coli* dominance in the gut of pigs associated with the occurrence of bacterial diarrhea on a farm.

In the case of farm occurrence of bacterial diarrhea, the model ignored the death of pigs, and two scenarios (metaphylaxis using colistin, and no use of colistin) were considered (Fig. 1). Regardless of diarrhea disease status, in pigs with *mcr*-harboring *E. coli* exhibiting a colistin MIC ≥ 4 μg/mL, at any concentration of *E. coli* in the gut, the colistin-resistant *E. coli* will be selected and become dominant, and they will remain dominant at a given maintenance probability (default: 80%) until the time of harvesting. This maintenance is a function of the unknown fitness conferred on *E. coli* by *mcr***-**harboring plasmid. In contrast, selection of plasmid-mediated colistin-resistant *E. coli* will not occur if the pigs do not have *mcr*-harboring *E. coli* in the gut. In the scenario in which therapeutic colistin is not used, regardless of disease status, the proportions of *mcr*-harboring colistin-resistant *E. coli* in dominating and non-dominating pigs and those that do not harbor the *E. coli* in the gut follow the field situation at farms without intensive colistin selection pressure due to treatment, which will be explained in more detail below.

**Fig. 2.**
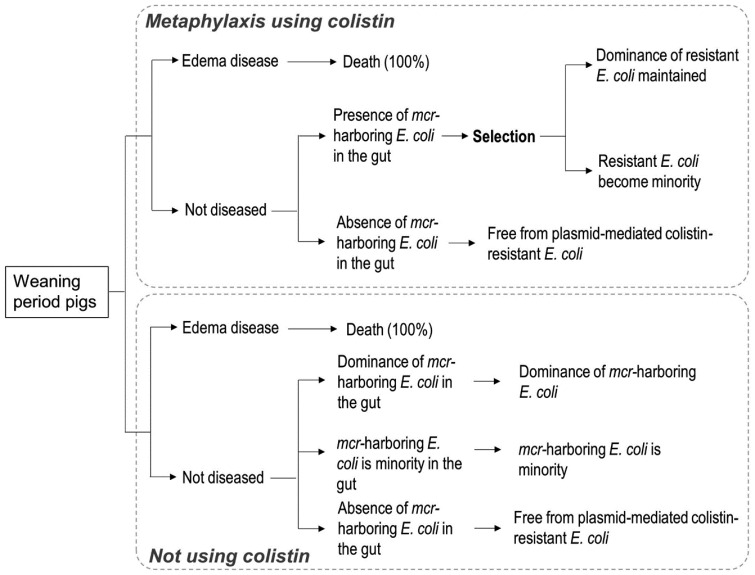
Flowchart for plasmid-mediated colistin-resistant *E. coli* dominance in the gut of pigs associated with the occurrence of edema disease on a farm.

Regarding edema disease occurrence on a farm, all of the diseased animals die in the model, and again, two scenarios (metaphylaxis or no use of colistin) were considered (Fig. 2). When metaphylaxis was used on a farm in which a proportion of pigs have *mcr*-harboring *E. coli* in the gut, all of the non-diseased pigs having *mcr*-harboring colistin-resistant *E. coli* at any concentration of *E. coli* will exhibit dominance of colistin-resistant *E. coli* in the gut. According to the function of the unknown fitness conferred on *E. coli* by *mcr*-harboring plasmid, a proportion of pigs in which *mcr*-harboring colistin-resistant *E. coli* was selected will continue to have resistant *E. coli* dominant in the gut. In the scenario in which therapeutic colistin is not used, pigs in which *mcr*-harboring colistin-resistant *E. coli* does and does not dominate and those that do not have this *E. coli* in the gut among non-diseased pigs follow the field situation at farms without intensive colistin selection pressure due to treatment, identical to bacterial diarrhea cases.

**Fig. 3.**
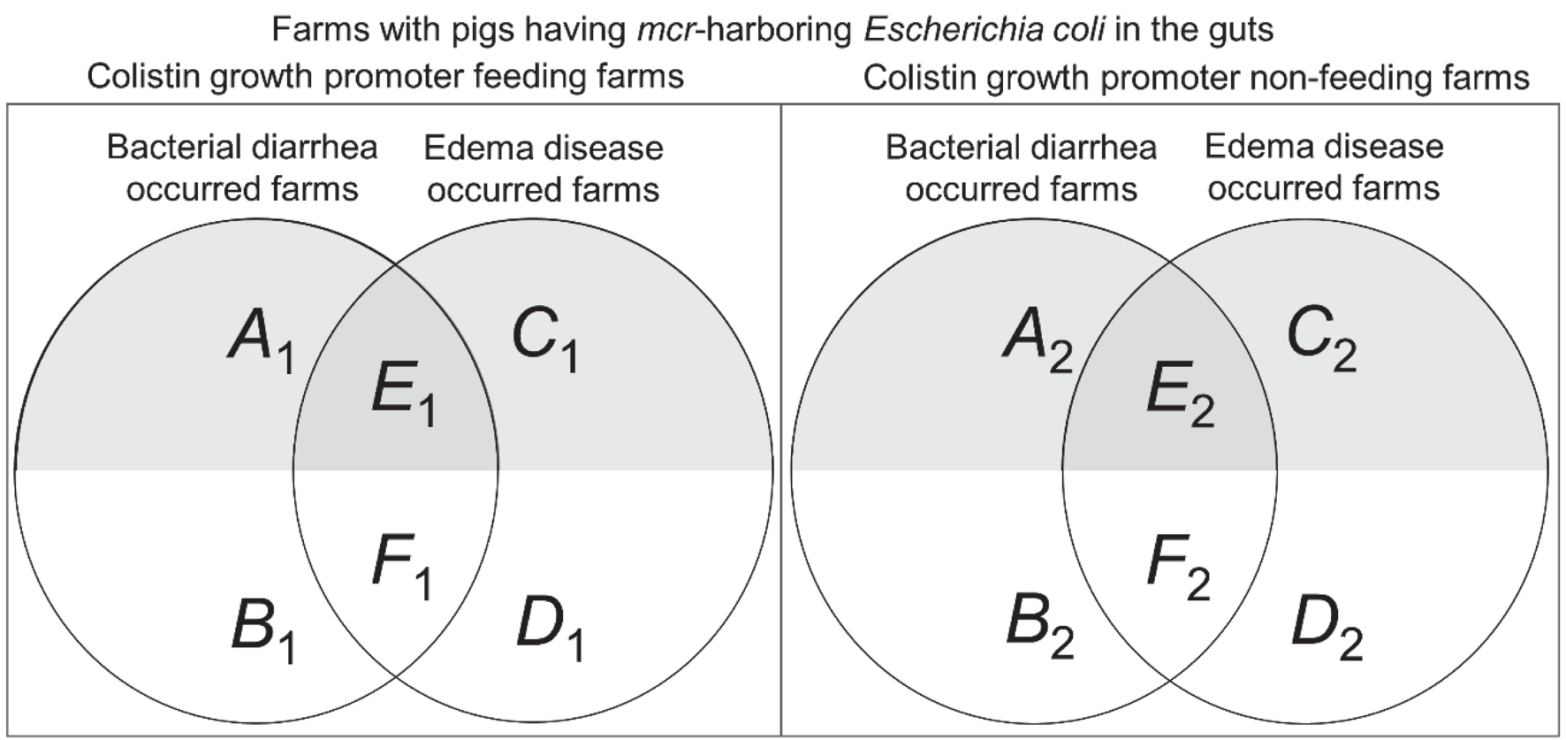
Venn diagram showing the categories of farms in terms of use of colistin as a growth promoter, occurrence of bacterial diarrhea and edema disease, and use of therapeutic colistin. Shaded areas indicate farms that used therapeutic colistin.

In the model, bacterial diarrhea and edema disease occur on randomly selected farms, and within these farms, based on the steps explained in Figs. 1 and 2, the number of pigs with *mcr*-harboring colistin-resistant *E. coli* dominant in the gut will be calculated. Fig. 3 shows the Venn diagrams for the categories of swine farms based on the use of colistin growth promoter (left and right panels), occurrences of bacterial diarrhea and edema disease (overlapped circles), and the use of therapeutic colistin (shaded and non-shaded areas within the circles). The areas *A_i_* and *C_i_* indicate the farms in which bacterial diarrhea and edema disease occur, respectively, and therapeutic colistin is used, where *i* = 1 represents farms at which colistin is used as a growth promoter; *i* = 2 represents non–colistin-feeding farms. The areas *B_i_* and *D_i_* are similar to *A_i_* and *C_i_*, and the difference is that therapeutic colistin is not used in these farms. On the farms in the areas *E_i_* and *F_i_*, both bacterial diarrhea and edema disease occur, and therapeutic colistin is used in the farms in areas *E_i_*, while not used in *F_i_*. The calculation is implemented in three separate farm-size categories, *j* (small, medium, and large) (equation 1). *A_ijres_* to *E_ijres_* in Equation 1 indicate the number of pigs in which plasmid-mediated colistin-resistant *E. coli* dominate in the gut among the farm categories A*i* to E*i* in Fig. 3, and the total number of finisher pigs with *mcr*-mediated colistin-resistant *E. coli* dominating in the gut among the 1,000 farms is denoted as *N_mcr_*. As shown in Fig. 3, both bacterial diarrhea and edema disease occur on farm *E_i_*, and double counting of plasmid-mediated colistin-resistant *E. coli*-dominant pigs occurs in this category. In contrast, double counting does not occur on farm *F_i_*, where therapeutic colistin is not used. To avoid double counting, the number of overlapping plasmid-mediated colistin-resistant *E. coli*-dominant pigs, *E_ijres_*, was deducted (Equation 1). Of all pigs born on the 1,000 farms, pigs with edema disease die, and the total number of pigs slaughtered, not including the number of pigs with edema disease, was calculated (*T_slaughtered_* in Equation 1). The risk of Japanese finisher pigs with *mcr*-mediated colistin-resistant *E. coli* dominating in the gut among all Japanese finisher pigs just prior to sending the animals to the slaughterhouse, on an arbitrary day, was calculated using Equation 1. The model was run for 5,000 iterations using the for-loop in R software. A sensitivity analysis was performed to ascertain the unknown probability of maintaining the dominance of *mcr*-*1-5*-mediated colistin-resistant *E. coli* in the gut of a pig after selection associated with therapeutic colistin use for the options of 40, 60, and 100% maintenance (default 80%). In the following sections, estimations of probability distributions of the variables used in the model are explained.

$$\text{Risk}=\frac{N_{mcr}}{T_{slaughtered}}=\frac{\sum_{j=1}^{3}\sum_{i=1}^{2}\left(A_{ijres}+B_{ijres}+C_{ijres}+D_{ijres}-E_{ijres}\right)}{T_{slaughtered}}$$

Equation 1

### 2.4. Estimation of the Proportion of Pigs with *mcr*-*1-5*-harboring Colistin-resistant *E. Coli* Dominant in the Gut on a Farm that Used Colistin as a Growth-promoting Feed Additive

If *mcr-1-5-*harboring colistin-resistant *E. coli* cultured from swine feces formed colonies that could be picked up on a non-selective bacterial agar, it was defined as being dominant in the gut. To determine the bacterial concentration in this situation, an experiment was carried out in early 2017 at four swine farms where colistin was used as a growth promoter (but not for therapeutic purposes) and there were pigs present with *mcr*-harboring *E. coli*. In the four farms, treatment using colistin had never been done before and during the experiment. Feces were sampled from 30 pigs, and three colonies of *E. coli* cultured on non-selective deoxycholate hydrogen sulfide lactose (DHL) agar were purified; isolates exhibiting an MIC ≥ 4 μg/mL were classified as colistin-resistant. When at least one *E. coli* colony was colistin-resistant, the pig of origin was classified as having colistin-resistant *E. coli* dominant in the gut (one qualitative result for each pig). The same samples were cultured using colistin-supplemented CHROMagar^TM^ COL-APSE (CHROMagar, Paris, France), and the *E. coli* concentration in feces was determined from the number of suspected colonies on the agar based on the color of the colonies (one quantitative result for each pig). Using the test results of 22 weaning-period or fattening pigs examined (colistin-resistant *E. coli* grew on colistin-supplemented CHROMagar for all of the 22 samples; the results of other suckling pigs and sows were not used in this analysis), receiver operating characteristic (ROC) analysis was performed to determine the cut-off value of the bacterial concentration to best differentiate between dominance of colistin-resistant *E. coli* in the gut or not, by maximizing both sensitivity and specificity using the ROCR package^16^^)^ in R software, version 3.5.1^15^^)^.

Under the uniform distribution, 100 pairs of non-selective DHL and CHROMagar results were randomly sampled from the results of the 22 pigs in 2017, and the proportion of samples exceeding the cut-off value was calculated. This process was iterated 5,000 times, and a beta distribution was fit to the simulated values to solve the parameters using the fitdist() function of the fitdistrplus package^17^^)^. This provided the probability, *P_dom_gp_*, that *mcr-1-5-*harboring *E. coli* would dominate in the gut of a pig on a farm that fed colistin as a growth promoter but did not use therapeutic colistin. The proportion of pigs with colistin-resistant *E. coli* dominance in the gut after therapeutic colistin use (*P_selected_gp_*) on a farm feeding colistin as a growth promoter was modeled using the beta distribution with the parameters specified by the number of *E. coli* samples that grew on colistin-supplemented CHROMagar at any bacteria concentrate, 22 of 22 samples (Table 2).

### 2.5. Estimation of the Proportion of Pigs with *mcr*-*1-5*-harboring Colistin-resistant *E. Coli* Dominant in the Gut on a Farm That Did Not Use Colistin as a Growth Promoter Feed Additive

As it was difficult to find farms not using colistin as a growth promoter feed additive, four farms that participated in the experiment described in section 2.4 and stopped use of colistin as a growth promoter immediately after the sampling in 2017 were studied again 12 months later. In both experiments in 2017 and 2018, 30 pigs each (in total 60 pigs) were used, and three *E. coli* isolates isolated from each feces sample (90 isolates in each year) cultured on DHL agar were tested for colistin resistance and *mcr-1-8*. The 1-year reduction rate in the animal-level prevalence of *mcr*-mediated colistin resistance (*Red_mcr_*) was calculated using equation 2.

$$Red_{mcr}=1-\frac{1-{\left(1-P_{mcr2018}\right)}^{3}}{1-{\left(1-P_{mcr2017}\right)}^{3}}$$

Equation 2 where *P_mcr_*_2017_ represents the proportion of *E. coli* isolates that were colistin-resistant and had any of *mcr-1* to *-8* in the 2017 experiment, and *P_mcr_*_2018_ represents that proportion in the 2018 experiment (all the colistin-resistant *E. coli* isolates had at least one of *mcr-1* to *-8*). In the simulation model, a point estimate of *Red_mcr_* was used, but for the purpose of presentation of the effect of stoppage, it was simulated stochastically separately using beta distributions. The reason we tested for *mcr-1* to *-8* rather than *mcr-1* to *-5* was that the objective of this experiment was different, and the results will be published elsewhere. It was assumed that the animal-level prevalence of *mcr-1-5-*harboring colistin-resistant *E. coli* dominating in the gut of pigs on a farm that never used colistin as a growth promoter would be similar to that observed 12 months after stoppage, as there was no actual relevant information available in Japan. The probability that *mcr-1-5-*harboring *E. coli* dominates in the gut of a pig on a farm that never fed growth promoter colistin or stopped feeding growth promoter colistin 12 months previously and had not used therapeutic colistin, *P_dom_nogp_*, was modeled by multiplying *P_dom_gp_* and a complement of *Red_mcr_* to 1 (Table 2). The proportion of pigs with colistin-resistant *E. coli* dominance in the gut after therapeutic colistin use on a farm that never fed growth promoter colistin or stopped feeding growth promoter colistin 12 months previously but did use therapeutic colistin (*P_selected_nogp_*) was modeled by multiplying *P_selected_gp_* and a complement of *Red_mcr_* to 1 (Table 2).

### 2.6. Estimation of the True Proportion of Japanese Farrow-to-finisher and Reproduction Swine Farms Having Pigs with *mcr*-harboring Colistin-resistant *E. Coli* in the Gut

**Fig. 4.**
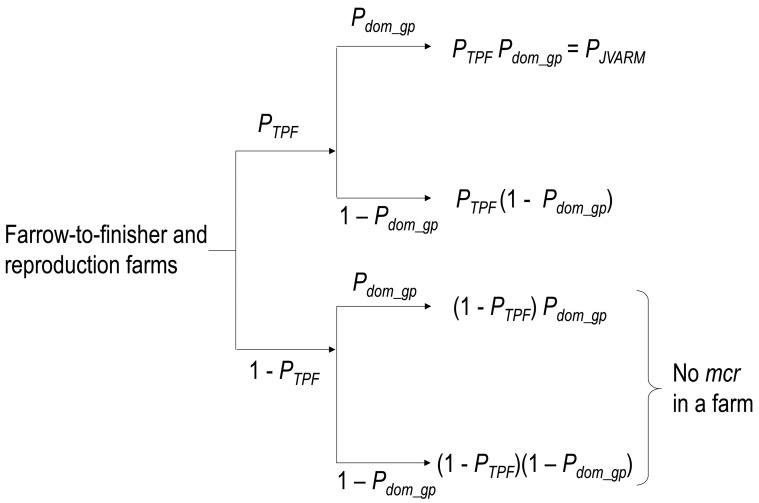
Diagram showing the probabilities associated with the true proportion of swine farms having pigs with *mcr-1-5-*harboring colistin-resistant *E. coli* in the gut (*P_TPF_*). *P_dom_gp_*: probability that *mcr-1-5-*harboring colistin-resistant *E. coli* is dominant in the gut of a pig; *P_JVARM_*: probability that colistin-resistant *E. coli* harboring *mcr-1-5* will be isolated from one sample of feces from a finisher swine just before harvesting on a farm in the sampling of the JVARM program.

Our study relies on the diagnosis of colistin resistance in *E. coli* by the JVARM, which collected only one sample from a farm; however, as described in the previous section, a proportion of negative samples might be collected from swine farms actually having pigs with *mcr-1-5-*harboring colistin-resistant *E. coli* in the gut. For this reason, the true proportion of Japanese farrow-to-finisher and reproduction swine farms having pigs with* mcr-1-5-*harboring colistin-resistant *E. coli* in the gut (*P_TPF_*) was estimated. The probability that colistin-resistant *E. coli* harboring *mcr-1-5* will be isolated from one sample of feces from a finisher swine just before harvesting on a farm in the sampling of the JVARM program, *P_JVARM_*, can be calculated as the product of *P_TPF_* and *P_dom_gp_* (Fig. 4). Therefore, *P_TPF_* is calculated using equation 3.

$$P_{TPF}=P_{JVARM}/P_{dom\_gp}$$

Equation 3

A previous report described the isolation of *mcr-1-5-*harboring *E. coli* exhibiting an MIC of 2 μg/mL^2^. In addition to the true farm-level prevalence of *mcr-1-5-*harboring colistin-resistant *E. coli*, the true prevalence of farms with pigs having *mcr*-harboring *E. coli* including susceptible ones (an MIC of 2 μg/mL) was estimated using Equation 3, but in this case, *P_JVARM_* indicated the proportion of fecal samples having *E. coli* isolates harboring *mcr-1-5* including susceptible isolates.

### 2.7. Estimation of 1-month Incidence Rates of Edema Disease and Bacterial Diarrhea Among Weaning Pigs at the Farm Level

Therapeutic colistin is used at the occurrence of edema disease or bacterial diarrhea, particularly during the 1-month weaning period. The incidence rates for edema disease and bacterial diarrhea at the farm level (*IR_dis k_*) were estimated separately and among different farm size categories (*k*) using results of the 2017 questionnaire for farms feeding colistin as a growth promoter and those of the 2018 questionnaire for farms not feeding colistin.

In the questionnaires, the number of farms falling into several categories of disease frequency (*l*) and size (*k*) were asked (Table 1). The number of farms in each category based on the veterinarian responses was summed to *n_lk_*. In modeling, for each farm size category (*k*), a set of *n_lk_* disease events within a 1-month period on farm *m* in disease frequency category (*l*) was drawn from a Poisson distribution with a rate parameter: the reciprocal of the between-disease-events period, which was drawn from a uniform distribution between *a_l_* and *b_l_* (e.g., 2 and 3 for the period category 2 to 3 months), was summed to calculate the total number of disease events occurring within a 1-month period in frequency category *l*. The number of disease events in the five disease frequency categories were summed and divided by the total number of farms in farm size category *k* (*T_Farm k_*) to obtain the 1-month incidence rate (*IR_dis k_*) for that farm size category (equation 4).

$$IR_{dis\;k}=\frac{\sum_{l=1}^{5}\sum_{m=1}^{n_{lk}}Poisson\left(n_{lk},\;\left(1/Uniform\left(a_{l},b_{l}\right)\right)\right)}{T_{Farm\;k}}$$

Equation 4

The calculation of *IR_dis k_* was iterated 5000 times, and a beta distribution was fit to the values using matching moment estimation in the fitdist() function of the R fitdistrplus package to obtain the posterior distribution. The number of farms in each of the disease frequency and size categories determined from the questionnaires are listed in Supplementary Tables 1 through 4.

### 2.8. Estimation of the Proportion of Weaning-period Pigs Affected by Edema Disease Or Bacterial Diarrhea within the Farm during An Occurrence

According to interviews with field swine veterinarians, at an occurrence of edema disease, almost 100% of the diseased pigs die, and in our model, these dead pigs must be excluded from the swine population. Moreover, in considering the mode of metaphylaxis (treating the entire herd or only affected pens), it is important to know the proportion of diseased weaning-period pigs at the occurrence of edema disease and bacterial diarrhea.

In the questionnaires, for edema disease and bacterial diarrhea and farm size categories separately, respondents were asked to allocate (to a total of 100%) weaning-period pigs affected by the disease into proportion categories, based on their clinical experience in 2017 and 2018 (Table 1). To estimate the proportion of affected pigs among weaning-period pigs on a farm, a proportion category for pigs affected was first selected, based on the weight given by the averaged percentage allocations of the categories using the sample() function in R. The random proportion was then assigned by drawing from a uniform distribution (*c*, *d*), where *c* represents the smaller range and *d* the larger range of the proportion category (e.g., for the 10.1-30% category, *c* = 10.1% and *d* = 30%). This process was iterated 5,000 times, and a beta distribution was fit to the sampled results using the fitdist() function in R, for 2017 and 2018 and different farm size categories. The weight matrixes used in these simulations are shown in Supplemental Tables 5 through 8. The distributions fit using 2017 data represented the situation in which colistin was used as a growth promoter, as 93% of farms were feeding colistin, whereas those fit using 2018 data represented the situation in which colistin was not used as a growth promoter.

### 2.9. Estimation of the Probability of Therapeutic Use of Colistin at the Occurrence of Edema Disease or Bacterial Diarrhea

In the questionnaires used in 2017 and 2018, respondents were asked to make point estimates of the probability of therapeutic use of colistin at the occurrence of edema disease or bacterial diarrhea (Table 1). For respective years, a set of 100 random samples from the pool of responses was drawn, and the mean was calculated. This process was iterated 5,000 times, and a beta distribution was fit to the means using the fitdist() function in R to calculate the probability of therapeutic colistin use given the indication disease occurred (*P_use|dis_*).

### 2.10. Assessment of the Effects of Stoppage of Growth Promoter Colistin Use and Shift of Colistin to a Second-choice Drug on the Occurrence of Indication Diseases and Frequency of Therapeutic Colistin Use

To compare the incidence rates of bacterial diarrhea and edema disease between 2017 and 2018, 50 samples each were drawn from the probability distributions of incidence rates for both years and logit transformed and compared using Welch’s *t*-test for both diseases. The sample size, 50, was determined by calculating the minimum sample size for a comparison of two means, so that the size exceeded the requirement for all farm size categories.

Even after shifting colistin from first-choice drug to second, if the frequency of indication diseases was increased, the frequency of therapeutic colistin use may not decline. Therefore, for bacterial diarrhea and edema disease, respectively, the probability of therapeutic colistin use in a given 1-month period on a farm of size category *k* (*P_use k_*) was calculated using Monte Carlo simulation of 5,000 iterations by multiplying the samples drawn from the posterior distributions of the 1-month incidence rate of disease (*IR_dis k_*) and probability of therapeutic colistin use at the occurrence (*P_use|dis_*) (equation 5). A set of 50 values was sampled from the posterior probability distributions of therapeutic colistin use, *P_use k_*, in a given 1-month period for 2017 and 2018, respectively, and logit transformed and compared using Welch’s *t*-test.

$$P_{use\;k}=IR_{dis\;k}\times P_{use|dis}$$

Equation 5

### 2.11. Scenario Analyses

Scenarios prepared for assessing potential intervention options included reduction of bacterial diarrhea and edema disease cases (50% and 80% reduction, respectively), reduction of the probability of therapeutic colistin use (50% and 80% reduction, respectively), reduction of the number of target pigs by pen-unit colistin use (20% of all weaning-period pigs therapy). For pen-unit use, the proportion 20% of all weaning pigs was chosen based on the proportion of diseased pigs at the occurrence of bacterial diarrhea or edema disease. The proportion of pigs with *mcr-1-5-*mediated colistin-resistant *E. coli* dominant in the gut after pen-unit therapy using colistin was calculated for farms where colistin as a growth promoter feed additive was used (*P_selected_pen_gp_*) and for farms where it was not used (*P_selected_pen_nogp_*), using equation 6 and 7, respectively.

$$P_{selected\_pen\_gp}=min\left\{0.2\times P_{selected\_gp}+\left(1-0.2\right)\times P_{dom\_gp},\;1\right\}$$

Equation 6

$$P_{selected\_pen\_nogp}=min\left\{0.2\times P_{selected\_nogp}+\left(1-0.2\right)\times P_{dom\_nogp},\;1\right\}$$

Equation 7

The primary purpose of this risk assessment was to characterize the risk of *mcr-1-5-*mediated colistin-resistant *E. coli* during a period of time when a majority of swine farmers were using colistin as a growth promoter. In addition, the risk at 12 months after stoppage of growth promoter colistin use and the shift of colistin to a second-choice therapeutic drug was assessed using the questionnaire survey results for 2018 on disease occurrence and therapeutic colistin use.

## 3. Results

### 3.1. Representativeness of Postal Survey Results

Of 82 members of the Japan Pig Veterinary Society, 28 (34.1%) responded in 2017, and 43 members (52.4%) responded in 2018. The number of farrow-to-finisher and reproduction farms for which information was collected was 294 in 2017 and 455 in 2018. The distributions of the farms studied by prefecture exhibited significant correlations between the number of farms studied and that registered in livestock census in both years (*ρ* = 0.78, *P* < 0.01 in 2017; *ρ* = 0.77, *P* < 0.01 in 2018, Fig. 5).

**Fig. 5.**
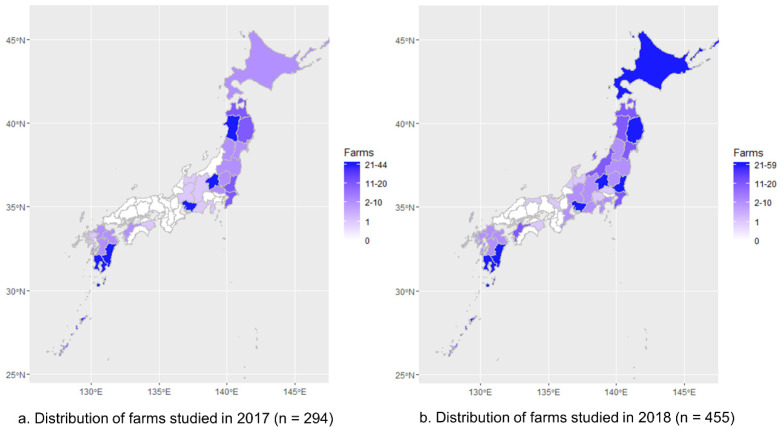
Distributions of farrow-to-finisher and reproduction farms for which information was collected in 2017 (left panel a, 294 farms) and 2018 (right panel b, 455 farms)

### 3.2. Within-farm Prevalence of Pigs with *mcr-1-5-*mediated Colistin-resistant *E. Coli* Dominant in the Gut

**Fig. 6.**
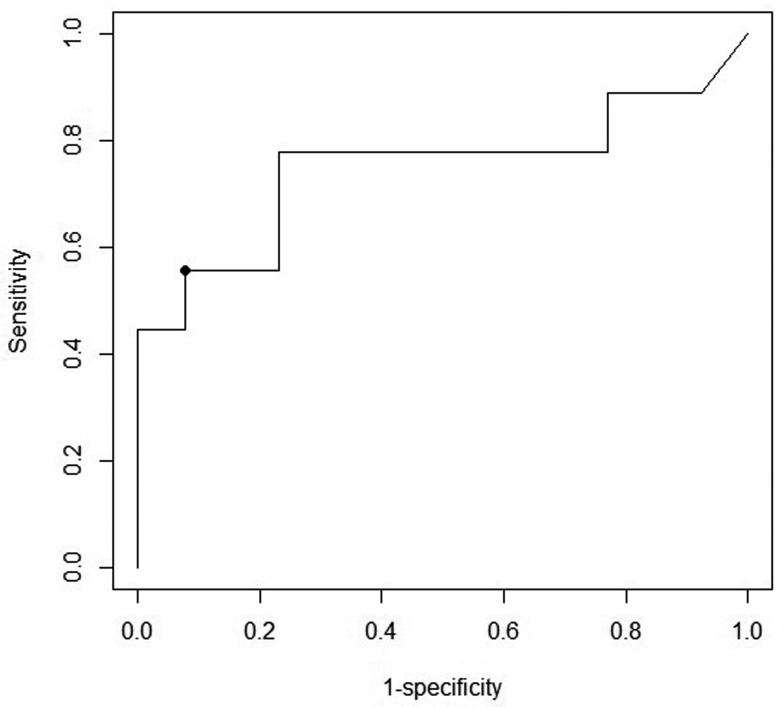
ROC curve prepared to determine the cut-off threshold of *mcr-1-5*-mediated colistin-resistant *E. coli* concentration for dominance in the swine gut. The point shows the cut-off threshold, 10^5.08^ CFU/g.

Table 2 shows the estimation results for variables associated with the within- and between-farm prevalence of *mcr-1-5-*mediated colistin-resistant *E. coli*. For the within-farm prevalence, the mean proportion of non-colistin-treated pigs with *mcr-1-5-*harboring colistin-resistant *E. coli* dominant in the gut in growth promoter colistin feeding farms (*P_dom_gp_*, 31.0% [95% credible interval, CI: 18.0%-45.6%, median 30.6%], Table 2) was estimated based on the dominance cut-off threshold of 10^5.08^ CFU/g, with an accuracy score of 0.77, sensitivity 55.6%, and specificity 92.3%, determined using ROC curve analysis (Fig. 6). In contrast, colistin-resistant *E. coli* was cultured from all 22 samples collected at 4 farms where *mcr-*harboring *E. coli* was detected in the range of 10^3^ to 1.12 × 10^8^ CFU/g on colistin-supplemented CHROMagar, and the probability of selecting resistant *E. coli* after therapeutic colistin use, in other words, the proportion of *mcr*-mediated colistin-resistant *E. coli* dominant pigs when therapeutic colistin was used, in growth promoter feeding farms (*P_selected_gp_*), was estimated to be 95.9% (95% CI: 85.2%-99.9%, median 97.0%, Table 2). In growth promoter colistin non-feeding farms, the proportion of *mcr*-mediated colistin-resistant *E. coli* dominant pigs was much lower: 13.1% (*P_dom_nogp_*, 95% CI: 7.6%-19.2%, median 12.9%) when therapeutic colistin was not used, and 40.5% (*P_selected_nogp_*, 95% CI: 36.0%-42.2%, median 40.9%) when it was used (Table 2).

### 3.3. Farm-level Prevalence of *mcr-1-5*-mediated Colistin-resistant *E. Coli* among Japanese Reproduction and Farrow-to-finisher Swine Farms

The true farm-level prevalence of pigs with *mcr-1-5-*harboring *E. coli* in the gut including susceptible ones (*P_TPF2_*), as in 2017 when growth promoter colistin was fed in majority of pig farms, was estimated to be 23.7% (95% CI: 13.9%-40.1%; median 22.6%), and that of plasmid-mediated colistin-resistant *E. coli* (*P_TPF_*) was estimated to be 15.5% (95% CI: 8.6%-26.5%; median 14.8%, Table 2).

### 3.4. Risk Estimation as of 2017

**Fig. 7.**
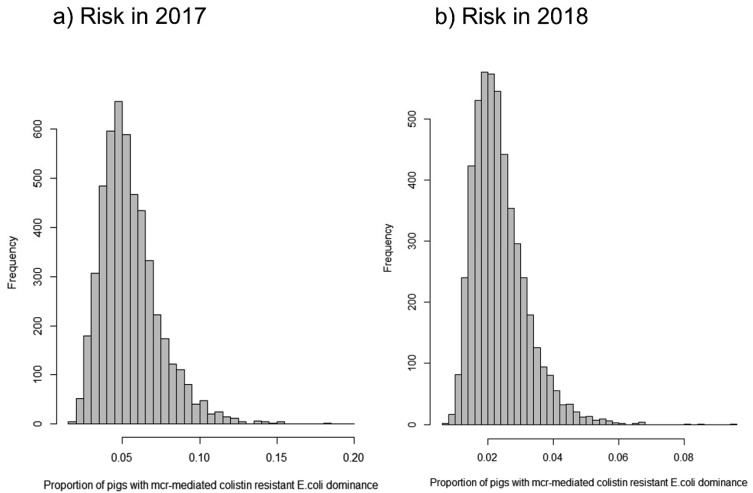
Distributions of the proportion of Japanese finisher pigs just prior to being sent to slaughterhouses with *mcr-1-5*-mediated colistin-resistant *E. coli* dominating in the gut in 2017 (panel a) and in 2018 (panel b).

The mean proportion of Japanese finisher pigs with *mcr-1-5*-mediated colistin-resistant *E. coli* dominating in the gut just prior to sending the animals to the slaughterhouse was estimated to be 5.5% (95% CI: 4.2%-10.1%; median 5.2%, Fig. 7a, Table 3) as of 2017, when colistin was fed to pigs as a growth promoter on 93% of farms, according to the results of the questionnaire survey. The risk was sensitive to the unknown probability of maintenance of colistin resistance in *E. coli* after selection due to therapeutic colistin use; a change in the probability of maintenance from 80% to 20% resulted in a 20.0% change ([5.5%-4.4%]/5.5%) in the mean overall risk (Table 3).

**Table 3.** Proportion of Japanese finisher pigs just prior to being sent to slaughterhouses with *mcr-1-5*-mediated colistin-resistant *E. coli* dominant in the gut as of 2017, at different probabilities of maintenance of resistance after selection associated with therapeutic colistin use (mean, median, and 95% credible interval)

Probability of maintenance of resistance	Overall	Small-scale farms	Medium-scale farms	Large-scale farms
20%	4.4%, 4.2% (3.3 – 8.3%)	4.6%, 4.4% (3.4 – 9.1%)	4.6%, 4.3% (3.4 – 8.8%)	4.3%, 4.1% (3.3 – 8.1%)
40%	4.8%, 4.5% (3.7 – 9.0%)	4.7%, 4.4% (3.4 – 9.2%)	4.8%, 4.5% (3.6 – 9.2%)	4.8%, 4.6% (3.7 – 9.0%)
60%	5.2%, 4.9% (3.9 – 9.8%)	4.7%, 4.4% (3.4 – 9.3%)	5.0%, 4.7% (3.7 – 9.5%)	5.3%, 5.0% (4.0 – 9.9%)
80% (Default)	5.5%, 5.2% (4.2 – 10.1%)	4.6%, 4.3% (3.3 – 9.0%)	5.2%, 4.8% (3.9 – 9.6%)	5.8%, 5.4% (4.3 – 10.5%)
100%	6.0%, 5.7% (4.6 – 11.1%)	4.8%, 4.4% (3.4 – 9.5%)	5.5%, 5.2% (4.1 – 10.3%)	6.3%, 6.0% (4.8 – 11.7%)

### 3.5. Effects of Stoppage of Growth Promoter Colistin Use and Shift of Colistin to a Second-choice Drug for Treatment

In the farm experiment, the proportion of *mcr*-mediated colistin-resistant *E. coli* among all *E. coli* isolates declined from 17.8% (16/90) in 2017 to 6.7% (6/90) in 2018. At the animal level, the mean reduction rate, *Red_mcr_*, was estimated to be 52.5% (95% CI: 8.7%-80.8%, median 54.8%, Table 2).

Table 4 shows the change between 2017 and 2018 in the 1-month incidence rates of bacterial diarrhea and edema disease in the weaning period (*IR_dis k_*). For both diseases, the overall rate increased significantly, and this change was due to the increased disease events on small- and medium-scale farms (*P* = 0.02 for small-scale farms, otherwise *P* < 0.01, Table 4). In contrast, the incidence rates for both diseases decreased significantly on large-scale farms (*P* < 0.01). The incidence rate was the lowest on small-scale farms and highest on large-scale farms in both years and for both diseases.

**Table 4.** Comparisons of 1-month incidence rates of bacterial diarrhea and edema disease in weaning-period pigs between 2017 and 2018 (*IR_dis k_*, n = 50 samples; mean, median and 95% credible interval)

Farm category	Year 2017	Year 2018	Statistics	*p*-value
***Bacteria diarrhea***				
Small-scale farms	3.1%, 2.4% (0.2% – 9.7%)	7.7%, 4.8% (0.1% – 30.8%)	t = 2.4, df = 191.6	0.02
Medium-scale farms	17.9%, 17.7% (12.3% – 24.3%)	26.7%, 26.6% (20.1% – 34.0%)	t = –12.3, df = 91.2	<0.01
Large-scale farms	40.1%, 40.1% (34.5% – 46.0%)	36.0%, 35.9% (30.0% – 42.1%)	t = 7.7, df = 97.3	<0.01
***Edema disease***				
Small-scale farms	0.5%, 0.1% (<0.1% – 3.0%)	6.7%, 4.8% (0.1% – 24.6%)	t = –2.9, df = 94.5	<0.01
Medium-scale farms	2.0%, 1.9% (0.6% – 4.3%)	5.6%, 5.4% (3.2% – 8.6%)	t = –10.5, df = 80.6	<0.01
Large-scale farms	10.2%, 10.2% (7.3% – 13.8%)	9.2%, 9.1% (6.0% – 12.9%)	t = 2.7, df = 85.6	<0.01

The mean probability of colistin use at the occurrence of bacterial diarrhea (*P_use|dis_*) declined slightly, from 37.3% (95% CI: 30.3%-42.5%, median 37.2%) in 2017 to 31.4% (95% CI: 26.1%-36.9%, median 31.4%) in 2018, and that of edema disease declined more markedly, from 55.0% (95% CI: 46.0%-63.7%, median 55.0%) in 2017 to 44.4% (95% CI: 36.9%-52.0%, median 44.4%) in 2018.

Table 5 shows comparisons of the probability of therapeutic colistin use in a given 1-month period, *P_use k_*. On small-scale farms, *P_use_* did not differ for bacterial diarrhea between 2017 and 2018 but increased significantly on medium-scale farms (*P* < 0.01) and decreased significantly on large-scale farms (*P* < 0.01) in 2018. *P_use_* for edema disease increased significantly on small- and medium-scale farms in 2018 (*P* < 0.01, respectively) but decreased significantly on large-scale farms (*P* < 0.01).

**Table 5.** Comparison of the probability of therapeutic colistin use in a given 1-month period between 2017 and 2018 (*P_use k_*, n = 50 samples; mean, median and 95% credible interval)

Farm category	Year 2017	Year 2018	Statistics	*p*-value
***Bacteria diarrhea***				
Small-scale farms	1.2%, 0.9% (0.1% – 3.6%)	2.4%, 1.5% (<0.1% – 9.4%)	t = –1.1, df = 95.8	0.26
Medium-scale farms	6.7%, 6.6% (4.4% – 9.4%)	8.4%, 8.3% (6.0% – 11.2%)	t = –5.1, df = 93.2	<0.01
Large-scale farms	15.0%, 14.9% (11.6% – 18.8%)	11.3%, 11.2% (8.6% – 14.1%)	t = 11.9, df = 97.9	<0.01
***Edema disease***				
Small-scale farms	0.3%, 0.1% (<0.1% – 1.6%)	3.0%, 2.1% (0.1% – 10.8%)	t = –8.3, df = 76.5	<0.01
Medium-scale farms	1.1%, 1.0% (0.3% – 2.4%)	2.5%, 2.4% (1.4% – 4.0%)	t = –10.1, df = 71.6	<0.01
Large-scale farms	5.7%, 5.6% (3.8% – 7.8%)	4.1%, 4.1% (2.6% – 5.9%)	t = 6.4, df = 95.9	<0.01

### 3.6. Scenario Analyses

Table 6 shows a comparison of the risks estimated between 2017 and 2018 considering the changes in disease occurrence, treatment patterns, and decline of the prevalence of plasmid-mediated colistin-resistant *E. coli* based on the farm experiments. In all farm size categories, the risk decreased by approximately one-half, and the overall risk in 2018 was estimated to be 2.3% (95% CI: 1.8%-4.3%, median = 2.2%; reduction rate = 58.2% [5.5% to 2.3%], Fig. 7b). However, the animal-level reduction rate of *mcr*-mediated colistin-resistant *E. coli* in previously colistin growth promoter feeding farms (*Red_mcr_*) had wide credible interval, and the overall risk in 2018 was estimated to be 1.0% (95% CI: 0.8%-1.8%, median = 0.9%) and 4.8% (95% CI: 3.7%-8.8%, median = 4.5%), when *Red_mcr_* took 80.8%, and 8.7%, respectively.

**Table 6.** Comparisons of the estimated proportion of pigs with *mcr-1-5*-mediated colistin-resistant *E. coli* dominant in the gut before being sent to slaughterhouses between 2017 and 2018 (mean, median and 95% credible interval)

Year	2017	2018
Overall	5.5%, 5.2% (4.2 – 10.1%)	2.3%, 2.2% (1.8 – 4.3%)
Small scale	4.6%, 4.3% (3.3 – 9.0%)	2.2%, 2.0% (1.6 – 4.2%)
Medium scale	5.2%, 4.8% (3.9 – 9.6%)	2.3%, 2.1% (1.7 – 4.2%)
Large scale	5.8%, 5.4% (4.3 – 10.5%)	2.4%, 2.2% (1.8 – 4.4%)

Table 7 shows the change in the proportion of finisher pigs with *mcr*-*1-5*-mediated colistin-resistant *E. coli* dominant in the gut by several intervention options using the 2017 model. Compared with the default scenario, which was estimation of the risk in 2017, the risk did not decline with the reduction in edema disease occurrence at the farm level. In contrast, an 80% reduction in the occurrence of bacterial diarrhea at the farm level reduced the overall mean risk by 9% ([5.5%-5.0%]/5.5%), and the reduction was greatest on large-scale farms (12% reduction [5.8%-5.1%]/5.8%). A decrease in the probability of therapeutic colistin use exhibited an even greater reduction; an 80% reduction in colistin use reduced the risk by 12.7% ([5.5%-4.8%]/5.5%). When the probability of therapeutic colistin use was not changed but pen-unit therapy was applied, an even greater reduction in risk was observed (14.5% reduction, [5.5%-4.7%]/5.5%), which exhibited an effect similar to stoppage of therapeutic colistin use (16.4% reduction to 4.6%). The reduction rate was greatest on large-scale farms, whereas the risk did not change on small-scale farms for all intervention options.

**Table 7.** Results of scenario analyses showing the proportion of finisher pigs with *mcr*-*1-5*-mediated colistin-resistant *E. coli* dominant in the gut using the 2017 model (mean, median and 95% credible interval)

Scenario	Overall	Small-scale farms	Medium-scale farms	Large-scale farms
Default	5.5%, 5.2% (4.2 – 10.1%)	4.6%, 4.3% (3.3 – 9.0%)	5.2%, 4.8% (3.9 – 9.6%)	5.8%, 5.4% (4.3 – 10.5%)
***Reduction of edema disease***				
50% reduction	5.5%, 5.2% (4.2 – 10.0%)	4.6%, 4.3% (3.3 – 9.0%)	5.1%, 4.8% (3.8 – 9.6%)	5.7%, 5.4% (4.4 – 10.4%)
80% reduction	5.5%, 5.2% (4.2 – 10.1%)	4.6%, 4.2% (3.3 – 9.0%)	5.0%, 4.7% (3.7 – 9.6%)	5.8%, 5.4% (4.4 – 10.5%)
***Reduction of diarrhea***				
50% reduction	5.2%, 4.9% (3.9 – 9.7%)	4.6%, 4.2% (3.3 – 8.9%)	4.9%, 4.6% (3.6 – 9.3%)	5.3%, 5.0% (4.1 – 9.9%)
80% reduction	5.0%, 4.7% (3.7 – 9.4%)	4.6%, 4.2% (3.3 – 8.9%)	4.8%, 4.5% (3.5 – 9.1%)	5.1%, 4.8% (3.8 – 9.7%)
***Reduction of therapeutic colistin***				
50% reduction	5.1%, 4.8% (3.9 – 9.4%)	4.6%, 4.3% (3.3 – 9.0%)	4.9%, 4.6% (3.6 – 9.2%)	5.2%, 4.9% (4.0 – 9.7%)
80% reduction	4.8%, 4.5% (3.6 – 9.2%)	4.6%, 4.3% (3.3 – 9.1%)	4.7%, 4.4% (3.5 – 9.1%)	4.9%, 4.5% (3.6 – 9.1%)
Stoppage of therapeutic use	4.6%, 4.3% (3.4 – 8.7%)	4.6%, 4.3% (3.3 – 9.1%)	4.6%, 4.3% (3.4 – 8.9%)	4.6%, 4.3% (3.4 – 8.8%)
Pen level treatment (20% of pigs)	4.7%, 4.4% (3.5 – 8.7%)	4.6%, 4.3% (3.3 – 9.0%)	4.7%, 4.4% (3.5 – 8.7%)	4.7%, 4.4% (3.6 – 8.7%)

The distributions of 1-month incidence rates, proportion of weaning pigs affected at occurrence of indication diseases, and probability of therapeutic colistin use that were used in the simulations are provided in Supplemental Table 9.

## 4. Discussion

This study used an individual-based model for quantitative release assessment of the selection of *mcr-1-5*-mediated colistin-resistant *E. coli* in Japanese pigs just before slaughtering associated with growth promoting and therapeutic uses of colistin. To the best of our knowledge, this is the first study in the world to have taken this approach.

The mean proportion of pigs with *mcr-1-5-*mediated colistin-resistant *E. coli* dominating in the gut just before slaughtering was estimated at 5.5% as of 2017, and *mcr* genes were assessed as being widely spread in Japan: approximately one-fourth (23.7%) of reproduction and farrow-to-finisher swine farms, including those that did not use colistin, were estimated to have pigs with *mcr*-harboring *E. coli*.

In this assessment, parameters of probability distributions were determined based on JVARM data, questionnaire surveys, and farm experimental data, and were not solved using observed JVARM data by fitting approaches such as maximum-likelihood estimate, Markov-Chain Monte Carlo simulation, or approximate Bayesian computation^18^^)^. Additional validation process may be needed for the model in future. However, the estimated risk was within the 95% CI of the proportion of positive samples for *mcr-1-5*-mediated colistin-resistant *E. coli* in the JVARM results (Table 2); thus, the model assumption is plausible. Moreover, the purpose of the assessment included evaluating potential intervention programs, which this study achieved.

However, the model has several limitations: (1) already reported *mcr-6-9*^6^^,^^7^^,^^8^^,^^9^^,^^10^^)^, and chromosomal-associated colistin resistance^4^^)^ were not considered; (2) information on edema disease and bacterial diarrhea was based on questionnaire surveys, and actual clinical records were not used; (3) the probability of maintenance of colistin resistance after selection remains unknown; (4) transmission of *mcr*-harboring *E. coli* or transmission of plasmid-harbored *mcr* genes between pigs, between pens, and between farms was not modeled; and (5) detailed within-farm hygiene practices were not modeled.

Regarding limitation (1) above, the actual risk associated with *mcr* is higher for the unknown proportion of *mcr-6* to *-10* that can cause colistin resistance in *E. coli*, and our assessment underestimated this risk. Chromosomal colistin-resistant *E. coli* does not transmit resistance to other bacteria and was therefore outside the scope of this study. However, future completion of testing for *mcr-6* to *-10* or the potential discovery of other novel *mcr* genes using JVARM *E. coli* isolates would enable re-evaluation of the *mcr* risk and even the risk associated with chromosomal colistin-resistant *E. coli* using our simulation model, as our model is designed to select colistin-resistant *E. coli* regardless of the type of resistance, whether plasmid mediated or chromosomal.

Regarding limitation (2), in addition to a lack of accurate information from clinical records, questions in the postal questionnaire survey of 2017 related to bacterial diarrhea were phrased to refer to “weaning-period diarrhea”. Some veterinarians suggested that the questions should have referred to “bacterial diarrhea”, as the focus of the study was colistin-resistant *E. coli*. In the questionnaire provided in 2018, 16 of 28 respondents who participated in the 2017 survey responded in 2018 as well. A half of the respondents (50.0%, 8/16) answered about bacterial diarrhea, and one respondent (6.3%, 1/16) included diarrhea of a cause other than bacterial in 2017, whereas seven (43.8%, 7/16) could not remember (results not shown). However, considering the increase in 1-month incidence among small- and medium-scale farms in 2018, it is unlikely that the incidence rate of bacterial diarrhea in 2017 was substantially overestimated. Moreover, even if our estimates of incidence rates were accurate, the change in incidence rate might have been due to factors other than risk management, such as pure variability (e.g. purely random variation of disease occurrence).

Analysis of the maintenance rate of colistin resistance after selection due to therapeutic use of colistin showed moderate sensitivity. In the United Kingdom, an outbreak of *mcr*-harboring colistin-resistant *E. coli* has been reported only on a pig farm, and by stopping therapeutic colistin use, *mcr* was eliminated from the farm after 20 months^19^^)^. In Spain, by reducing therapeutic colistin use, the proportion of positive samples for colistin-resistant *Salmonella* in swine feces declined from 60% in 2015 to 35% in 2017, and that for *mcr-1* in feces also declined, from 70% in 2015 to 53% in 2017^20^^)^. According to our farm experiment estimate, by stopping the use of colistin as a growth promoter in feed, 52.3% of pigs with *mcr*-mediated colistin-resistant *E. coli* dominance in the gut would lose colistin-resistant *E. coli* in 12 months.

Biologically, both the transmission and maintenance of *mcr* genes are affected by the type and size of the host plasmid^18^^)^. Therefore, our risk estimate of the post–risk management situation in 2018 is sensitive to variability in the characteristics of plasmids harboring *mcr* genes, which was not considered in the simulation model. To understand the dynamics of within-farm clearance of *mcr* genes, the relationship between the full genome sequence of *mcr*-harboring plasmids and the speed of clearance should be studied, and mathematical modeling could be suitable for this purpose, as it has been applied to model transmission elsewhere^21^^)^.

Scenario analyses provided several clear insights. First, stoppage of colistin use as a growth promoter may be the most effective means of reducing the risk of producing pigs with *mcr*-mediated colistin-resistant *E. coli* dominant in the gut. Second, controlling bacterial diarrhea and reducing therapeutic colistin use have instantaneous effects on risk reduction, although the degree of reduction is not particularly high when compared with stoppage of colistin use as a growth promoter. Comparing the results of the questionnaire surveys for 2017 and 2018 showed reductions in both the incidence of bacterial diarrhea on large-scale farms and therapeutic colistin use in 2018. Pig veterinary clinicians appeared to respond well to the change by the implementation of risk management. Third, limited use of therapeutic colistin for affected pens was more effective than reducing the therapeutic use of colistin in entire weaning pig herds by 80%. According to the interviews with pig veterinary clinicians, metaphylaxis involving colistin administration via feed tanks was the most common mode, and the default model takes this option. As *mcr* genes pose health risks in humans, selective and pludent colistin use would reduce these risks in Japan more rapidly.

This study involved only release assessments at pig farms. The qualitative risk assessment conducted by FSCJ described the risk pathways for transmission of *mcr* genes to MDRP, MDRA, and CRE in the human gut via foods contaminated with *mcr*-harboring bacteria^22^^)^. Colistin is the first choice for treating infections with MDRP, MDRA, or CRE, but it will not work if these pathogens have obtained *mcr* genes. More detailed experiment-based information related to the transmission of *mcr* genes between bacteria within the human gut and the associated clinical consequences is needed. In the future, it would be worthwhile to conduct a complete quantitative risk assessment of colistin resistance.

In conclusion, the mean probability of releasing pigs with *mcr-1-5-*mediated colistin-resistant *E. coli* dominant in the gut to slaughterhouses in Japan was estimated to be 5.5% in 2017 and 2.3% in 2018, after stoppage of use of colistin as a growth promoter and shifting therapeutic colistin to second-choice drug. Scenario analyses confirmed that these risk management options were well targeted. Pen-unit treatment and reduction of bacterial diarrhea via hygiene improvements, including the use of *E. coli* vaccines^23^^)^, would further reduce the risk. Monitoring of *mcr*-mediated colistin-resistant bacteria in pigs should be continued, and whole-genome sequencing of *mcr*-harboring plasmids would provide more-accurate knowledge that could be used to further reduce the risk of *mcr*-mediated colistin-resistant bacteria in Japan.

## Supplemental Tables

**Supplemental Table S1.** Number of farms fallen in bacterial diarrhea frequency and farm size categories in 2017 questionnaire survey

Number of sows	>1/month	Once per 2-3 months	Once per 4-6 months	Once per 7-12 months	Once per 2-3 years	Almost no occurrence	Total
=<50	0	0	0	1	0	4	5
51-100	1	12	8	5	2	38	66
101-200	8	17	6	13	3	30	77
201-500	12	12	6	4	3	18	55
>500	27	10	8	4	15	11	75
Total	48	51	28	27	23	101	278

**Supplemental Table S2.** Number of farms fallen in bacterial diarrhea frequency and farm size categories in 2018 questionnaire survey

Number of sows	>1/month	Once per 2-3 months	Once per 4-6 months	Once per 7-12 months	Once per 2-3 years	Almost no occurrence	Total
=<50	0	0	1	6	0	4	11
51-100	4	15	25	18	0	10	72
101-200	11	29	16	22	6	15	99
201-500	21	39	27	16	3	32	138
>500	25	33	26	8	0	16	108
Total	61	116	95	70	9	77	428

**Supplemental Table S3.** Number of farms fallen in edema disease frequency and farm size categories in 2017 questionnaire survey

Number of sows	>1/month	Once per 2-3 months	Once per 4-6 months	Once per 7-12 months	Once per 2-3 years	Almost no occurrence	Total
=<50	0	0	0	0	0	5	5
51-100	0	2	0	1	1	62	66
101-200	1	0	4	1	0	71	77
201-500	0	5	2	0	4	44	55
>500	9	2	3	1	8	52	75
Total	10	9	9	3	13	234	278

**Supplemental Table S4.** Number of farms fallen in edema disease frequency and farm size categories in 2018 questionnaire survey

Number of sows	>1/month	Once per 2-3 months	Once per 4-6 months	Once per 7-12 months	Once per 2-3 years	Almost no occurrence	Total
=<50	0	0	5	0	1	10	16
51-100	0	0	6	4	2	56	68
101-200	4	3	6	8	6	72	99
201-500	2	12	10	5	5	100	134
>500	4	15	6	8	4	66	103
Total	10	30	33	25	18	304	420

**Supplemental Table S5.** Averaged percentage allocations on the proportion of weaning period pigs diseased at the occurrence of bacterial diarrhea in clinical cases as of 2017

Number of sows	Proportion of weaning pigs affected						Total
	<10%	10.1-30%	30.1-50%	50.1-70%	70.1-90%	90.1-100%	
=<50	75.0	25.0	0	0	0	0	100
51-100	69.6	27.9	2.5	0	0	0	100
101-200	70.0	25.5	4.5	0	0	0	100
201-500	60.2	18.5	13.3	1.3	6.7	0	100
>500	48.1	37.8	4.7	1.2	8.2	0	100

**Supplemental Table S6.** Averaged percentage allocations on the proportion of weaning period pigs diseased at the occurrence of bacterial diarrhea in clinical cases as of 2018

Number of sows	Proportion of weaning pigs affected						Total
	<10%	10.1-30%	30.1-50%	50.1-70%	70.1-90%	90.1-100%	
=<50	0	0	31.3	0	6.2	62.5	100
51-100	0	0	8.8	5.9	2.9	82.4	100
101-200	4.0	3.0	6.1	8.1	6.1	72.7	100
201-500	1.5	9.0	7.5	3.7	3.7	74.6	100
>500	3.9	14.5	5.8	7.8	3.9	64.1	100

**Supplemental Table S7.** Averaged percentage allocations on the proportion of weaning period pigs diseased at the occurrence of edema disease in clinical cases as of 2017

Number of sows	Proportion of weaning pigs affected						Total
	<10%	10.1-30%	30.1-50%	50.1-70%	70.1-90%	90.1-100%	
=<50	75.0	25.0	0	0	0	0	100
51-100	83.8	16.2	0	0	0	0	100
101-200	67.0	28.0	5.0	0	0	0	100
201-500	64.3	11.0	22.7	1.3	0.7	0	100
>500	63.5	15.3	13.5	1.8	2.4	3.5	100

**Supplemental Table S8.** Averaged percentage allocations on the proportion of weaning period pigs diseased at the occurrence of edema disease in clinical cases as of 2018

Number of sows	Proportion of weaning pigs affected						Total
	<10%	10.1-30%	30.1-50%	50.1-70%	70.1-90%	90.1-100%	
=<50	100	0	0	0	0	0	100
51-100	94.1	5.9	0	0	0	0	100
101-200	80.8	14.2	4.0	1.0	0	0	100
201-500	82.9	10.0	5.0	2.1	0	0	100
>500	76.7	18.4	2.0	2.9	0	0	100

**Supplemental Table S9.** Parameters and distributions used for disease occurrence and therapeutic colistin use in the risk model

Parameter	Statistical distribution	Mean (Median)	95% CI	Source
***Bacterial diarrhea 1-month incidence rate* (*IR_dis k_*)**				
Small-scale farms which fed colistin growth promoter	Beta(1.387,43.623)	3.1% (2.4%)	0.2-9.7%	Questionnaire in 2017
Small-scale farms which did not feed colistin growth promoter	Beta(0.688,8.288)	7.7% (4.8%)	0.1-30.8%	Questionnaire in 2018
Medium-scale farms which fed colistin growth promoter	Beta(28.128,128.691)	17.9% (17.7%)	12.3-24.3%	Questionnaire in 2017
Medium-scale farms which did not feed colistin growth promoter	Beta(41.237,113.062)	26.7% (26.6%)	20.1-34.0%	Questionnaire in 2018
Large-scale farms which fed colistin growth promoter	Beta(111.600,166.215)	40.1% (40.1%)	34.5-46.0%	Questionnaire in 2017
Large-scale farms which did not feed colistin growth promoter	Beta(86.945,154.822)	36.0% (35.9%)	30.0-42.1%	Questionnaire in 2018
***Proportion of pigs affected by bacterial diarrhea in a farm at an outbreak***				
Small-scale farms	Beta(1.185,12.660)	8.7% (6.6%)	0.4-20.5%	Questionnaire in 2017
Medium-scale farms	Beta(0.866,6.476)	11.7% (8.4%)	0.2-41.2%	Questionnaire in 2017
Large-scale farms	Beta(111.600,166.215)	20.3% (10.8%)	<0.01-82.8%	Questionnaire in 2017
***Edema disease one-month incidence rate* (*IR_dis k_*)**				
Small-scale farms which fed colistin growth promoter	Beta(0.345,70.463)	0.5% (0.1%)	<0.01-3.0%	Questionnaire in 2017
Small-scale farms which did not feed colistin growth promoter	Beta(0.907,12.414)	6.7% (4.8%)	0.1-24.6%	Questionnaire in 2018
Medium-scale farms which fed colistin growth promoter	Beta(4.295,210.557)	2.0% (1.9%)	0.6-4.3%	Questionnaire in 2017
Medium-scale farms which did not feed colistin growth promoter	Beta(14.960,254.329)	5.6% (5.4%)	3.2-8.6%	Questionnaire in 2018
Large-scale farms which fed colistin growth promoter	Beta(35.170,305.776)	10.2% (10.2%)	7.3-13.8%	Questionnaire in 2017
Large-scale farms which did not feed colistin growth promoter	Beta(24.601,242.539)	9.2% (9.1%)	6.0-12.9%	Questionnaire in 2018
***Proportion of pigs affected by edema disease in a farm at an outbreak***				
Small-scale farms	Beta(1.185,12.660)	8.6% (6.6%)	0.4-27.5%	Questionnaire in 2017
Medium-scale farms	Beta(0.288,1.243)	18.4% (6.4%)	<0.01-84.8%	Questionnaire in 2017
Large-scale farms	Beta(0.360,1.453)	20.6% (9.7%)	<0.01-83.3%	Questionnaire in 2017
***Probability of therapeutic colistin use* (*P_use|dis_*)**				
At occurrence of bacterial diarrhea in 2017	Beta(66.339,111.587)	37.2% (37.3%)	30.3-42.5%	Questionnaire in 2017
At occurrence of bacterial diarrhea in 2018	Beta(88.762,193.777)	31.4% (31.4%)	26.1-36.9%	Questionnaire in 2018
At occurrence of edema disease in 2017	Beta(67.058,54.922)	55.0% (55.0%)	46.1-63.7%	Questionnaire in 2017
At occurrence of edema disease in 2018	Beta(73.076,91.425)	44.4% (44.4%)	36.9-52.0%	Questionnaire in 2018
